# Breaking Bias: The Role of Artificial Intelligence in Improving Clinical Decision-Making

**DOI:** 10.7759/cureus.36415

**Published:** 2023-03-20

**Authors:** Chris Brown, Rayiz Nazeer, Austin Gibbs, Pierre Le Page, Andrew RJ Mitchell

**Affiliations:** 1 Internal Medicine, Jersey General Hospital, St Helier, JEY; 2 Cardiology, Jersey General Hospital, St Helier, JEY

**Keywords:** human factors, chatgpt, medical errors, cognitive bias, clinical artificial intelligence

## Abstract

This case report reflects on a delayed diagnosis for a 27-year-old woman who reported chest pain and shortness of breath to the emergency department. The treating clinician reflects upon how cognitive biases influenced their diagnostic process and how multiple missed opportunities resulted in missteps. Using artificial intelligence (AI) tools for clinical decision-making, we suggest how AI could augment the clinician, and in this case, delayed diagnosis avoided.

Incorporating AI tools into clinical decision-making brings potential benefits, including improved diagnostic accuracy and addressing human factors contributing to medical errors. For example, they may support a real-time interpretation of medical imaging and assist clinicians in generating a differential diagnosis in ensuring that critical diagnoses are considered. However, it is vital to be aware of the potential pitfalls associated with the use of AI, such as automation bias, input data quality issues, limited clinician training in interpreting AI methods, and the legal and ethical considerations associated with their use.

The report draws attention to the utility of AI clinical decision-support tools in overcoming human cognitive biases. It also emphasizes the importance of clinicians developing skills needed to steward the adoption of AI tools in healthcare and serve as patient advocates, ensuring safe and effective use of health data.

## Introduction

Chest pain is a common reason for emergency department presentations, and delayed, or missed diagnosis can result in severe consequences for the patient. In this case report, we reflect on the clinician's diagnostic process and the influence of cognitive biases and human factors in assessing a 27-year-old woman who presented with chest pain and shortness of breath.

## Case presentation

We present the case of a 27-year-old woman who reported to the emergency department with sudden onset left-sided sharp pleuritic chest pain, which was attenuated on sitting forward. This was associated with shortness of breath without fever, cough, or hemoptysis. She had had no recent periods of immobility, illness, trauma, or surgery. There was no significant medical history; her only medication was the combined oral contraceptive pill. She was a non-smoker.

**Figure 1:**
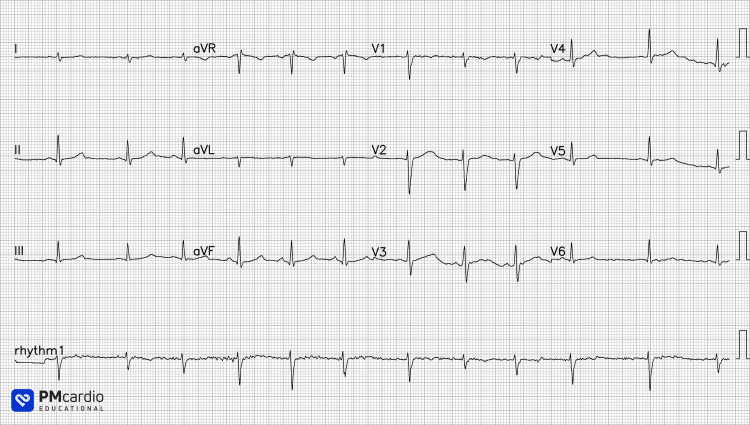
The patient's electrocardiogram is interpreted as sinus rhythm with possible electrical alternans. Paper ECG digitized using PMcardio (Powerful Medical; Bratislava, Slovakia).

**Figure 2:**
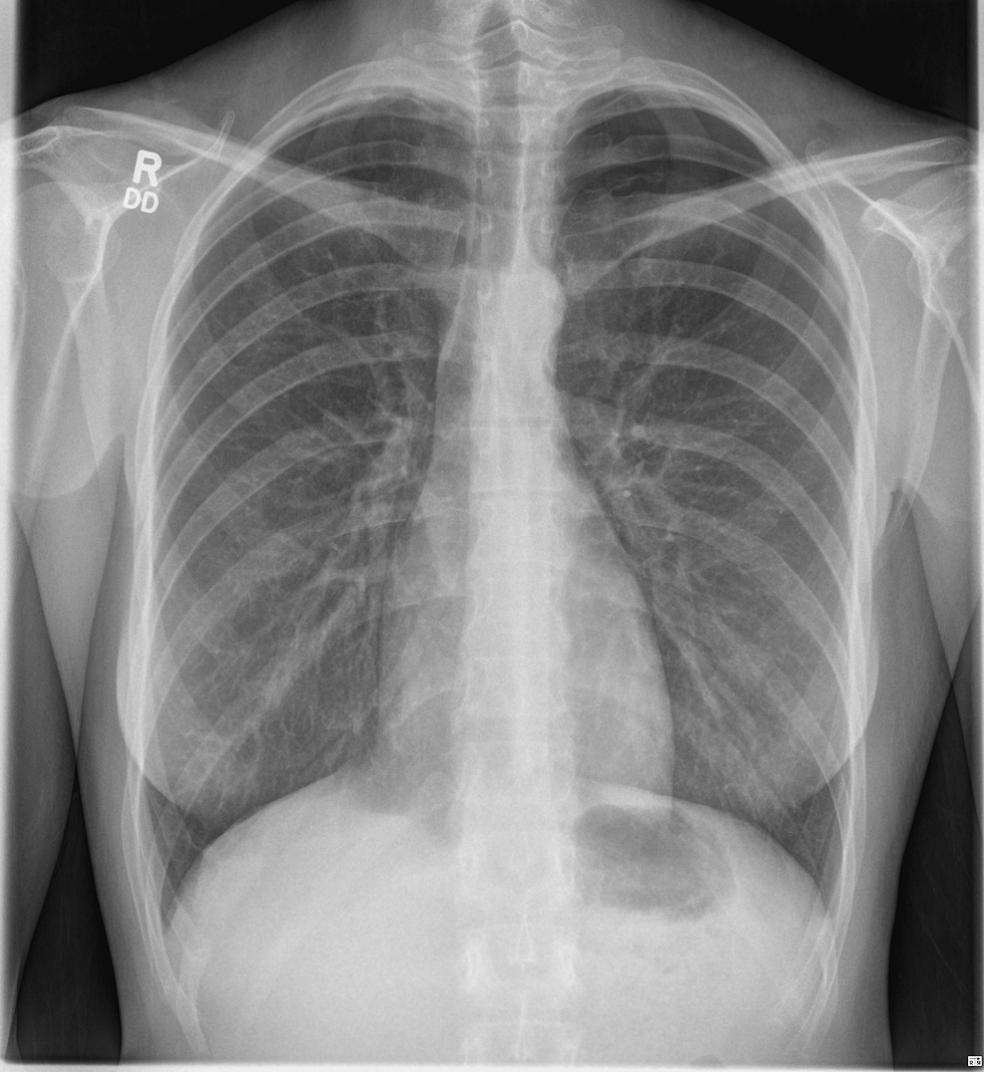
Patient’s chest radiograph, interpreted as showing no abnormality.

Her vital signs were normal on examination: she was not tachycardic or tachypnoeic, and oxygen saturations were 99% on room air. She was tall and slender, chest expansion was symmetrical, and air entry was described as equal throughout the auscultation of the chest. She had no leg swelling or calf tenderness. An electrocardiogram was performed and interpreted as sinus rhythm with electrical alternans, with periodic, beat-to-beat variation in QRS complex amplitude (Figure 1). A chest radiograph was performed, which was interpreted to show no acute abnormality (Figure 2).

The description of the pain being relieved by sitting forward and the finding of electrical alternans led to a suspicion of pericarditis with associated pericardial effusion. A point-of-care echocardiogram showed no evidence of pericarditis, pericardial effusion, or signs of right heart strain. The most likely differential was now perceived to be a pulmonary embolism, supported by a positive D-dimer test. The patient was anticoagulated and admitted under the medical team with a plan made for Computed Tomography (CT) Pulmonary Angiography the following day to confirm the diagnosis.

**Figure 3:**
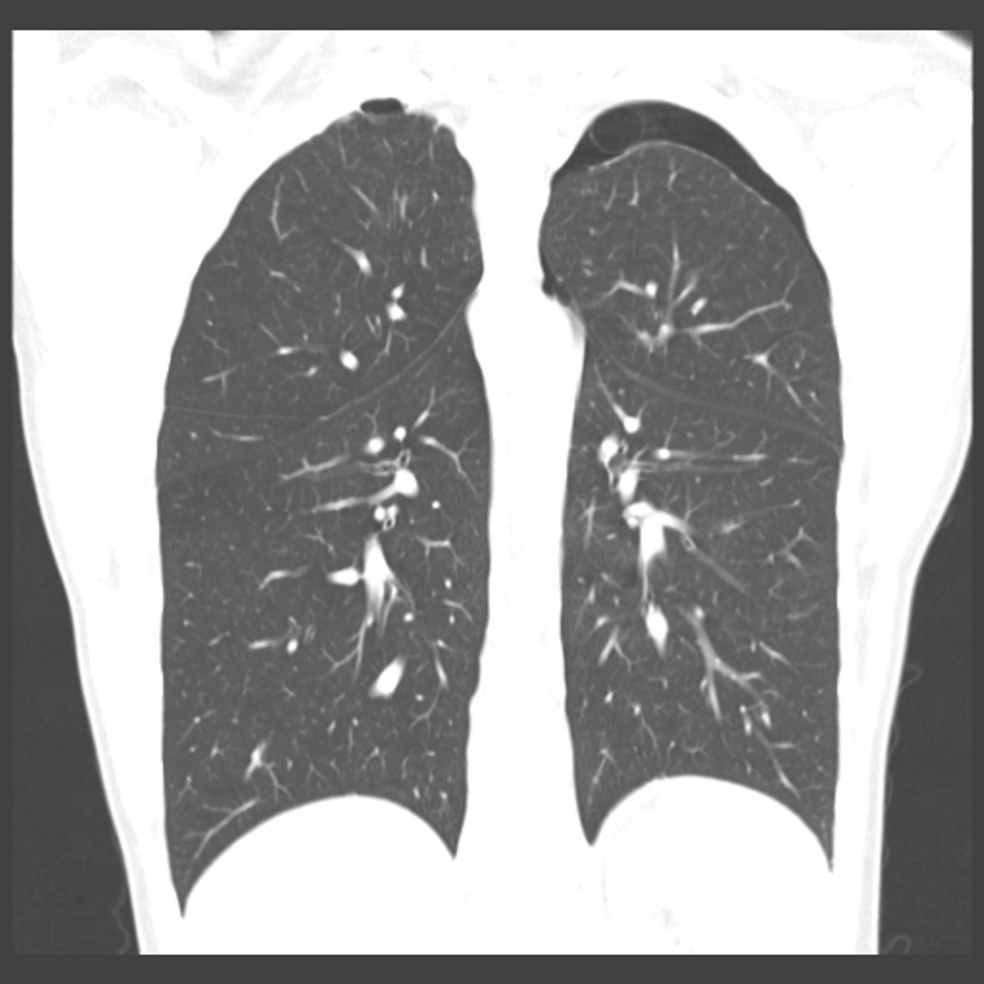
Patient's high-resolution chest computed tomography showing bilateral minor apical bullae and a small left-sided pneumothorax.

By the following morning, the radiologist's report for the chest radiograph was available, with a left apical pneumothorax measuring three centimeters identified. A subsequent high-resolution CT of the chest revealed bilateral apical bullae and persistent left-sided apical pneumothorax. This was managed conservatively in the acute setting. Subsequently, the patient underwent a Video-Assisted Thoracic Surgery (VATS)-guided bullectomy and pleurodesis, with a chest drain placed for the residual pneumothorax (Figure 3).

## Discussion

Clinician’s reflection

Human cognitive biases are common in medical practice and refer to systematic errors or deviations from rationality that may affect clinical decision-making [1]. Biases can be influenced by various factors, including clinical experience, environmental factors, and cognitive processes [2]. Unmitigated, these biases can lead to missed or delayed diagnoses, incorrect treatment plans, and adverse patient outcomes.

In the emergency department, multiple additional factors can negatively affect decision-making, such as time constraints, pressure, frequent task switching, and high cognitive load, making these settings among the highest risk in medicine [3]. This case exemplifies how multiple cognitive biases can compound, leading to delayed or inaccurate diagnoses. As we describe the patient's journey to diagnosis, we identify multiple vital points where these biases influence the clinician's diagnostic process.

In the emergency department, due to the need to identify critical cardiac problems, clinicians are often handed an ECG to interpret without the accompanying clinical context. In this case, the emergency physician first reviewed the ECG and identified possible electrical alternans before assessing the patient.

Electrical alternans on ECG (alternating QRS complex amplitude in any lead, due to the 'swinging heart') is an ECG sign suggestive of pericardial effusion, which is frequently found in association with pericarditis, while typical ECG findings of acute pericarditis - such as widespread ST-segment elevation - can be missing in up to two-fifths of cases [4]. The history and examination of this patient were subsequently framed with this prior suspicion of pericardial effusion. This framing may have biased the history taken, influencing the patient's descriptions of the pain: a pleuritic pain that was relieved by sitting up and leaning forward, consistent with classical pericarditic pain. Furthermore, there was an element of confirmation bias during the assessment with echocardiography. While the rationale for performing point-of-care ultrasound was to exclude pericardial effusion, the difficulty in obtaining echocardiography windows in a young patient with thin habitus was unexpected but overlooked. A pneumothorax could have impaired echocardiographic windows, and a fuller assessment of the chest with ultrasound may have revealed the true diagnosis [5].

The influence of human factors on clinical decision-making should be considered. This patient was one of many seen by this clinician at the end of a busy shift with a high-acuity case mix. There is often a call for frequent task-switching and interruptions due to an emergency department's unpredictable demands. The need to frequently make decisions quickly with often limited information can also lead to decision fatigue with limited cognitive resources [3].

In addition, these diagnostic missteps continued even as responsibility for care was passed from the emergency clinician to the inpatient medical team. Whilst the medical team reviewed the chest radiograph, again the pneumothorax was overlooked, perhaps due to the diagnostic momentum of pulmonary embolism. This diagnostic momentum finally ended with the radiologist's interpretation of the chest radiograph, made with only the brief clinical history ('Pleuritic chest pain, sudden onset. On OCP. Pain radiating to the left side of neck and shoulder') and without any framing from the treating clinicians' suspected diagnoses. It was fortunate in this case that the report was available promptly within 12 hours of the study being performed, meaning that the patient ultimately received the appropriate management and did not come to significant harm from delayed diagnosis.

What Do Medical Errors Have to Do with Swiss Cheese?

**Figure 4:**
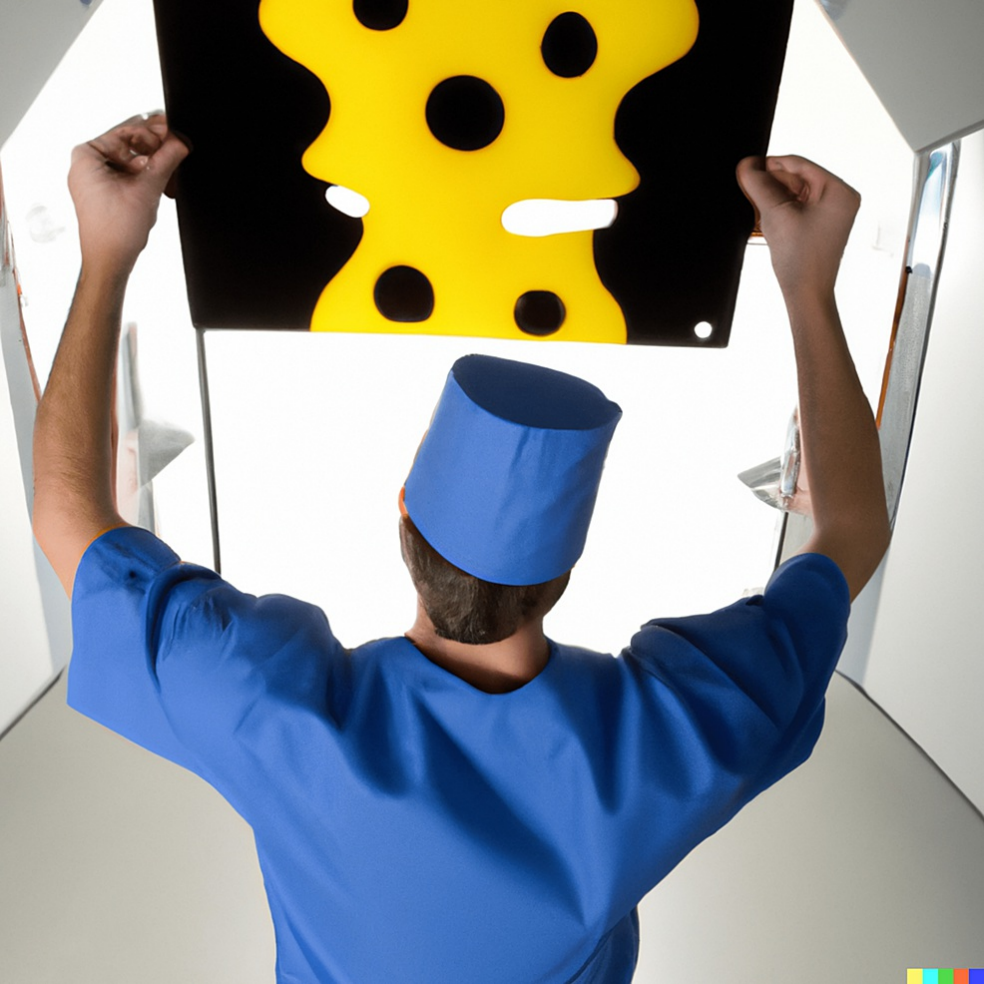
‘Swiss Cheese’: AI-generated image using DALLE-2 (OpenAI; San Francisco, USA) showing “A wide angle shot from behind of doctor in scrubs holding up an X-ray of a cartoon swiss cheese.”

Medical errors often culminate in multiple contributing factors, with multiple missed opportunities to curtail or prevent errors resulting in misdiagnosis or patient harm. The 'Swiss Cheese' model, or cumulative acts effect, is frequently applied to healthcare to illustrate how errors occur due to multiple safeguards failing in succession (figure 4) [6]. In this case, although these initial opportunities were missed even as responsibility for care was passed to the inpatient medical team, the safeguard of having formal reporting of all x-rays was the critical intervention that ultimately prevented patient harm.

Modern medicine's reliance on a growing range and volume of medical imaging has outpaced the expansion of the radiologist workforce. As a result, the Royal College of Radiologists has raised concerns about considerable variation and significant backlogs in imaging reporting, recognizing that serious patient harm due to delayed diagnosis has occurred [7]. Formal reports by specialist radiologists are rarely available in time to aid the early management of an acutely ill patients, particularly outside of regular working hours.

Artificial intelligence - the ability of machines to perform tasks that would typically require human intelligence - may provide innovative approaches to supporting clinicians amongst increasing healthcare demand and workforce pressures. The adoption of AI tools in radiology has been facilitated by the presence of Picture Archiving and Communication Systems, which provide a centralized platform for digital imaging and make it easier to integrate AI, in contrast to other medical specialties where health data is often fragmented across multiple systems that are not well-integrated or interoperable [8].

AI tools can offer a near real-time interpretation of medical imaging and clinical decision support and may identify latent patterns that may not be evident to clinicians. While humans are prone to cognitive biases, such as prejudice or fatigue, which can hinder their decision-making process, AI can mitigate these biases and improve accuracy in patient care.

However, AI is not (yet) the panacea. AI can learn and perpetuate bias in its training data, and it can be challenging to elicit how an AI makes its predictions, with these tools often described as a 'black box' [9]. A collaborative approach between clinicians and AI may provide the best possible outcome for patients by combining the unique advantages of AI pattern recognition and human contextual interpretation.

An alternative reality: AI augmenting and safeguarding clinical decision-making

**Figure 5:**
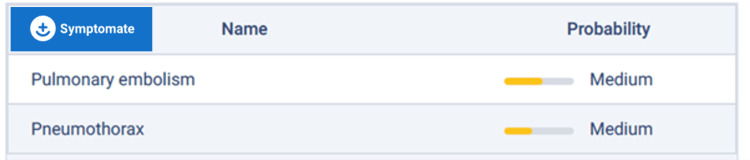
Possible diagnoses predicted by AI-based symptom checker tool Symptomate (Infermedica; Wrocław, Poland) using this patient’s presenting symptoms.

Before attending the emergency department, the patient could have used an AI-based symptom checker, such as Symptomate (Infermedica; Wrocław, Poland), to identify possible diagnoses and suggest where to seek help. Inputting this patient's symptoms results in a recommendation to call an ambulance due to possible conditions being pulmonary embolism or pneumothorax (Figure 5).

Similarly, after documenting the clinical history in an electronic health record (perhaps assisted by an AI scribe), AI tools could extract key features using natural language processing and with other parameters such as physiological observations and biomarkers, can support clinicians by generating a differential diagnosis, ensuring that critical diagnoses are considered, and identifying patients at higher risk of deteriorating [10,11]. At the hospital level, routine clinical data collected from a patient's arrival at the emergency department could be used to predict the patient's risk of needing admission, assisting with patient flow and service planning [12].

Clinician interpretation of investigations can be augmented using AI tools specific to the investigation - or multimodal AI tools capable of combining data from multiple inputs. AI has the potential to provide a more personalized interpretation of blood results and identify correlations and patterns unique to each individual. At the same time, human clinicians generally rely on standard reference ranges for interpretation [13].

**Figure 6:**
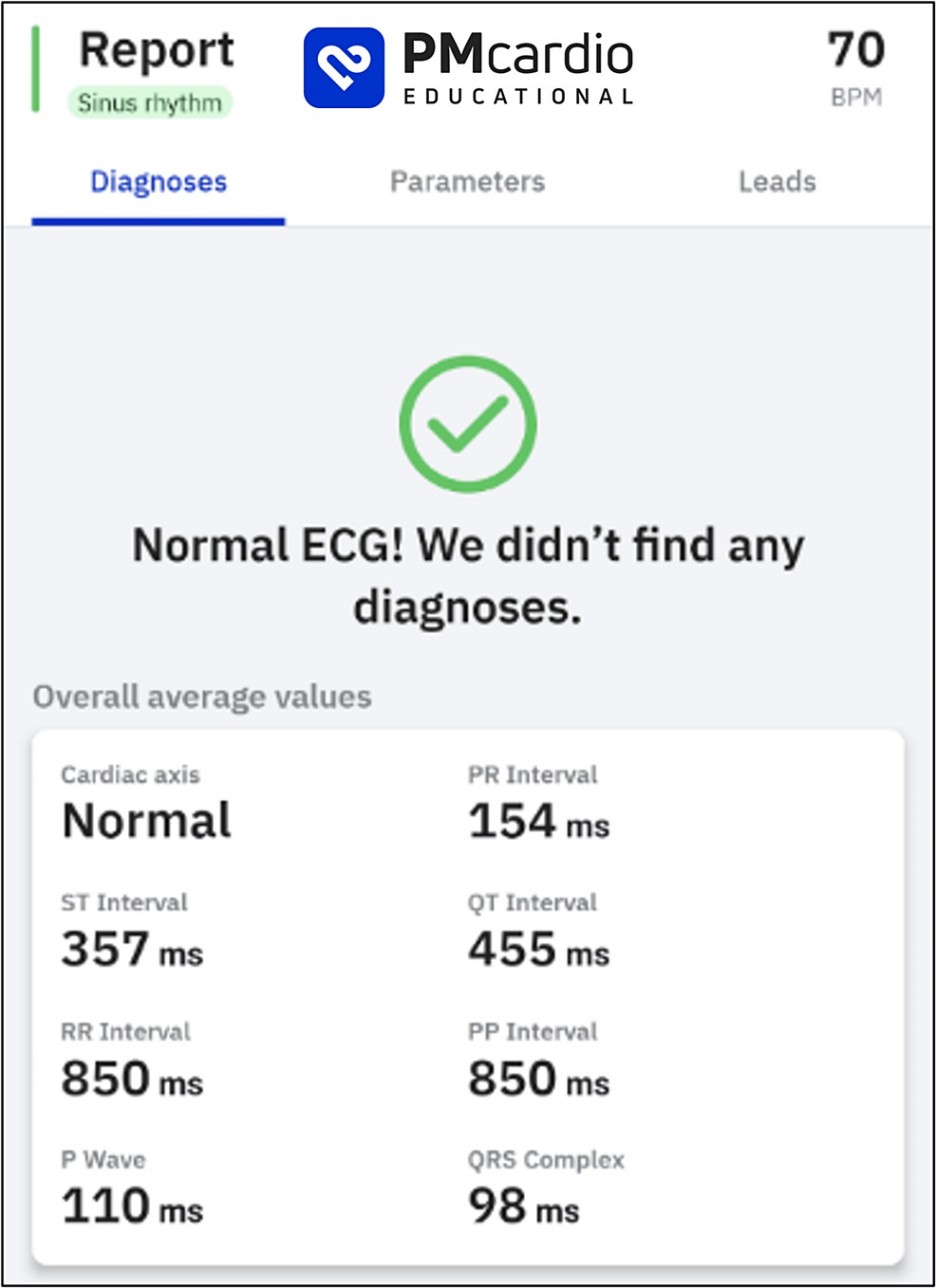
AI interpretation of this patient’s electrocardiogram using PMcardio (Powerful Medical; Bratislava, Slovakia).

**Figure 7:**
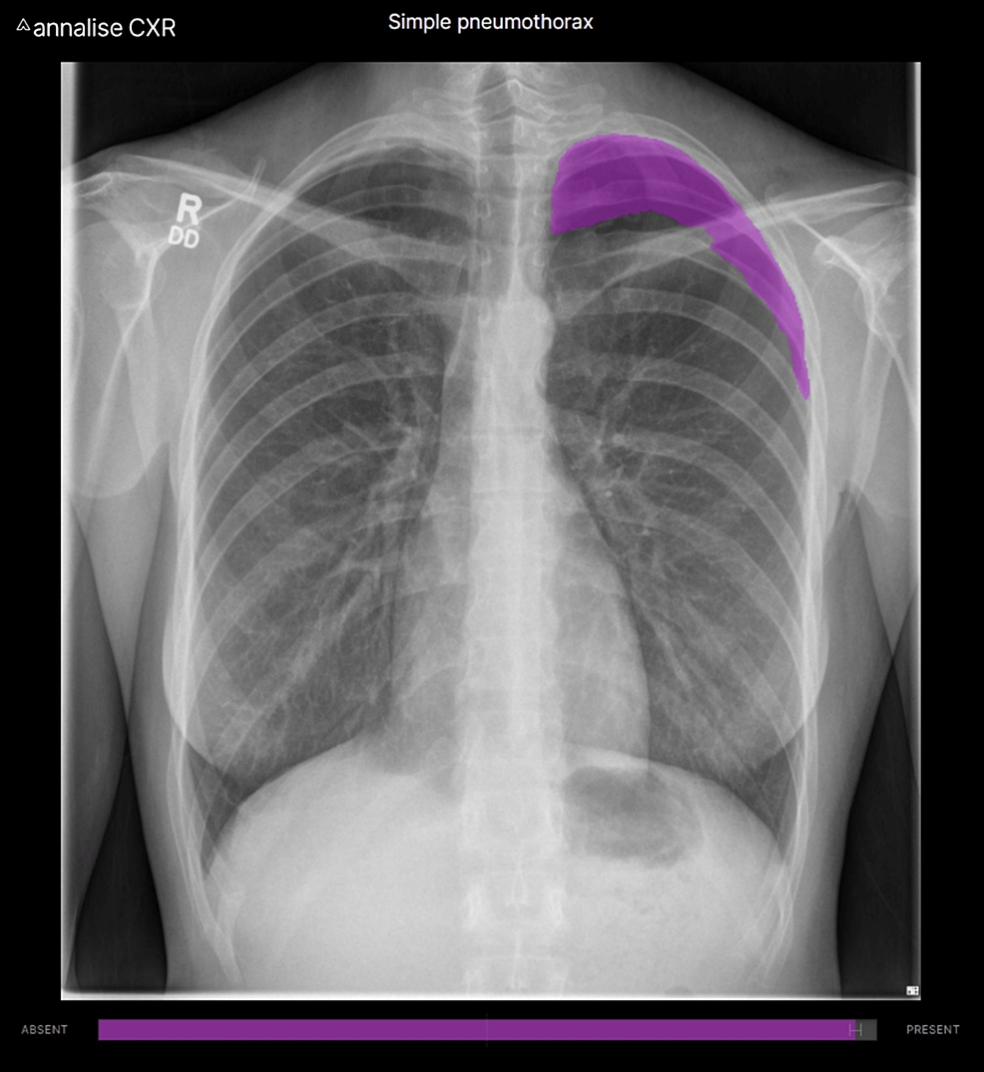
This patient’s chest radiograph displayed within Annalise Enterprise CXR (Annalise; Sydney, Australia), with AI-identified finding of pneumothorax overlaid.

Increasingly, AI tools are available to augment clinician diagnoses that rival or better the accuracy of expert clinicians in their fields. For example, using PMcardio (Powerful Medical; Bratislava, Slovakia), a paper ECG can be digitized for AI processing and instantly screened for 38 cardiovascular diseases (Figure 6). Many different AI models have been developed to detect pathology on chest x-rays. These range from disease-specific models (such as in early detection of SARS-CoV2) to more comprehensive solutions, such as Annalise Enterprise CXR (Annalise; Sydney, Australia), which can identify 124 distinct radiographic findings (Figure 7).

Had such tools been available, it is difficult to imagine that this patient's pneumothorax could have been missed initially. While there was no patient harm in this case, anticoagulation has risks, and appropriate management was potentially delayed.

AI: An imperfect companion to an imperfect clinician

While AI can help clinicians avoid cognitive biases, it is vital to be aware of the potential pitfalls associated with its use:

Overreliance on AI

Overreliance on AI systems and the assumption that they are infallible or less fallible than human judgment - automation bias - can lead to errors. Clinicians must remain critical and maintain due scrutiny to identify where the AI tool is flawed or makes incorrect predictions [14].

Biased AI

AI systems may be biased, reflecting biases in the data on which they are trained or in the algorithms used to build them [15]. Clinicians must be aware of these biases and take steps to ensure that AI is not reinforcing or perpetuating them.

Data Quality

The data quality of training an AI system can significantly affect its performance. The AI system may make correct predictions or recommendations if the data is complete, accurate, biased, and generalizable across diverse healthcare settings.

Interpretation of Results

Clinicians must be able to interpret the results generated by AI systems accurately. This requires an understanding of the underlying algorithms and statistical methods used by the system, as well as an awareness of the limitations of the data and the system itself [16,17].

Legal and Ethical Considerations

Legal and ethical implications are associated with using AI in clinical practice, particularly regarding privacy and informed consent issues. Automated decision-making is also a key issue in using AI in clinical practice, particularly in cases where AI systems are used to make decisions that could impact a patient's health. The General Data Protection Regulation (GDPR) places strict requirements on automated decision-making, including the right of individuals to obtain an explanation of the logic involved in automated decision-making and the right to challenge such decisions [18]. Clinicians must ensure that their use of AI in clinical decision-making is transparent and that patients receive adequate explanations of the decisions made by the AI system.

Liability

The responsibility for the care and treatment of a patient ultimately rests with the clinician, and they must take appropriate precautions when using AI systems to ensure that their decisions are based on the best available evidence and expertise. At the same time, organizations and individuals involved in developing and implementing AI systems are also responsible for ensuring that the systems are accurate, reliable, free from bias, and used responsibly and ethically.

## Conclusions

This case is an example of where clinical AI could have improved clinical decision-making and reduced the risk of patient harm. As with many medical advancements, clinicians have had to adapt their practice to incorporate new diagnostics and therapeutics. Just as the microscope's invention and the X-ray's discovery allowed clinicians to see the human body in a new way, AI tools and platforms can reveal new insights and patterns in health data, benefiting the patient in front of us and at the population level. Clinicians must develop the skills to ensure that AI tools are used effectively, safely, and in our patient's best interests. Ongoing real-world evaluation of clinical AI is essential to ensure that promise from pre-clinical data is borne out in reality. 

The potential of AI to revolutionize healthcare is unmistakable, yet we must remain cognizant of its limitations and the role of human clinicians in medicine. Though AI systems can contribute to improved diagnostics, personalized treatment plans, and predictive analytics, they cannot substitute the human qualities of empathy, adaptability, and ethical awareness. Complex decision-making, practical communication skills, and legal accountability are integral components of patient care that AI has yet to master. Moreover, AI's reliance on data quality and its current technological constraints demand human oversight to guarantee optimal patient outcomes. As such, we should envision AI as a powerful ally to augment and support clinicians rather than a replacement, fostering a synergistic relationship between technology and human expertise to improve healthcare delivery and patient well-being.

## Figures and Tables

**Figure 8:**
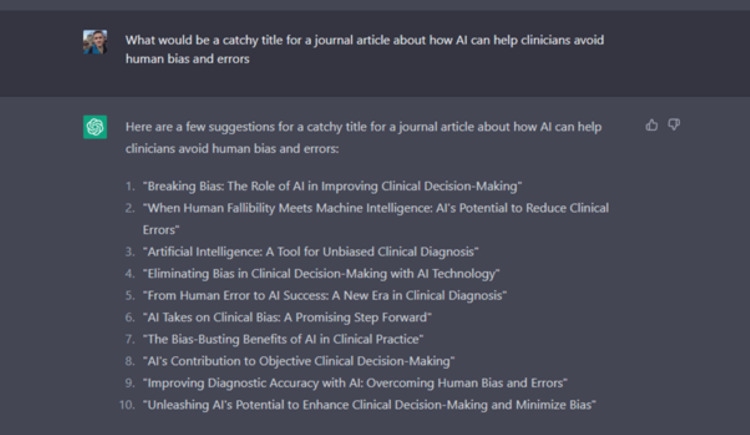
Using ChatGPT to brainstorm case report title ideas.

**Figure 9:**
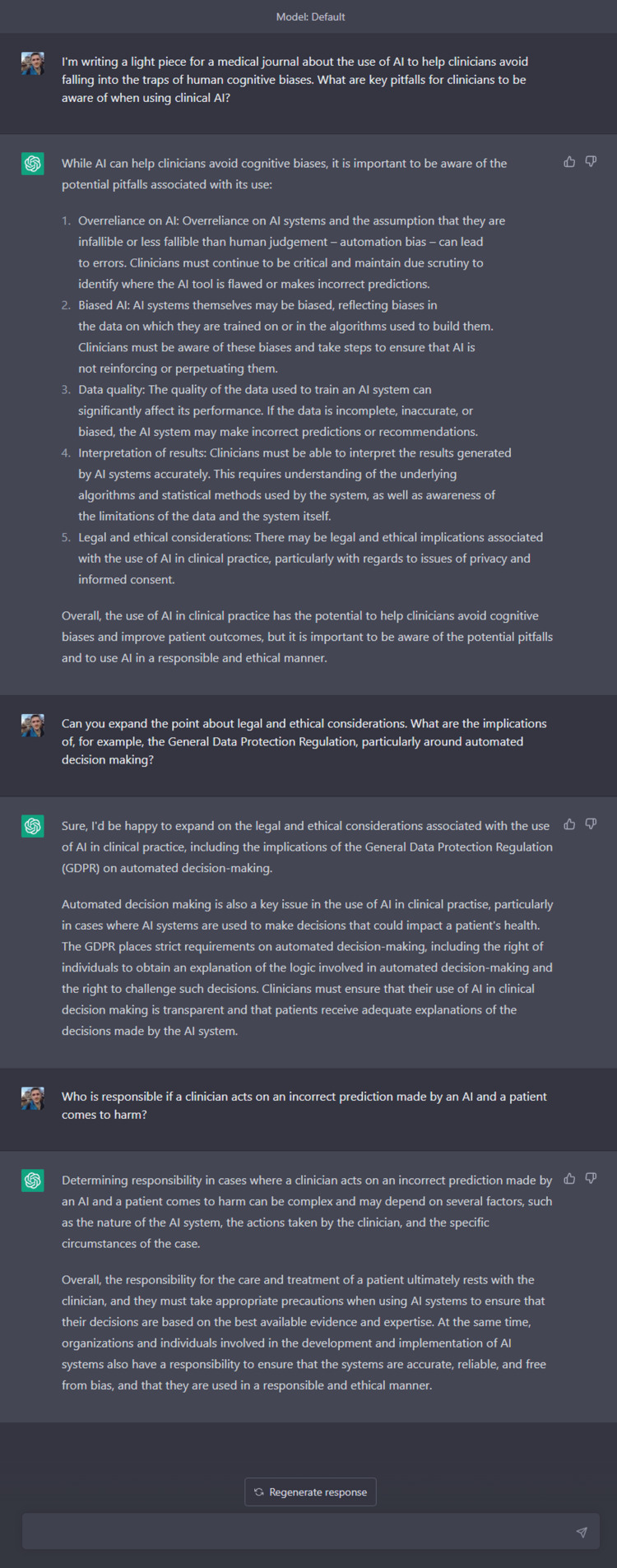
Using an AI tool (ChatGPT) to identify pitfalls of using AI tools in healthcare settings.

**Figure 10:**
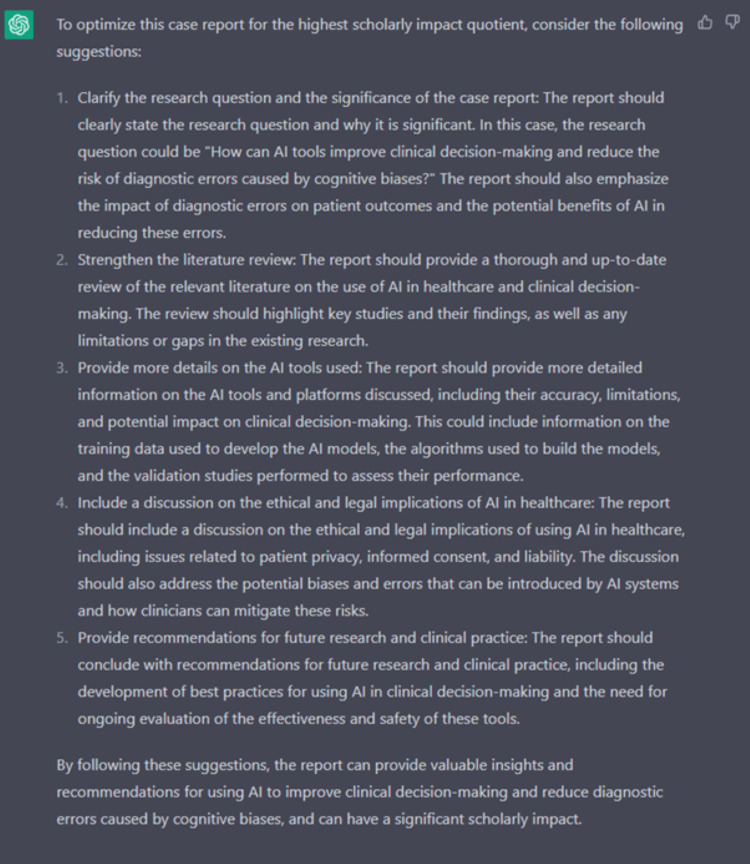
Using ChatGPT to critique drafts and suggest improvements.
